# A report of laryngeal adenocystic carcinoma metastatic to the spleen and the role of splenectomy in the management of metastatic disease: a case report

**DOI:** 10.1186/1752-1947-4-207

**Published:** 2010-07-06

**Authors:** Bryce W Murray, Lewis C Lyons, Anne T Mancino, Sergio Huerta

**Affiliations:** 1 Dallas VA Medical Center, Surgical Services (112), 4500 S Lancaster Road, Dallas, TX 75216, USA; 2 University of Arkansas for Medical Sciences, 4301 W Markham, Little Rock, AR 72205, USA

## Abstract

**Introduction:**

Adenoid cystic carcinoma (ACC) of the larynx is a rare malignancy characterized by an indolent course and late pulmonary metastases. Metastases from the larynx to the spleen are an unusual event. In the present report, we discuss a patient with adenoid cystic carcinoma of the larynx metastatic to the spleen. A review of the literature did not yield any other such incidents. We review the clinical presentation and course of adenoid cystic carcinoma, as well as the role of splenectomy for metastases.

**Case presentation:**

We present a case of laryngeal adenoid cystic carcinoma in a 26-year-old Caucasian man treated with total laryngectomy and ionizing radiation. He initially developed asynchronous pulmonary metastases, which were resected. Our patient subsequently presented with a symptomatic splenic lesion consistent with metastatic disease, for which he underwent laparoscopic splenectomy.

**Conclusions:**

Splenectomy might be indicated for isolated metastases. A splenectomy effectively addresses symptoms and serves as a cytoreduction modality.

## Introduction

Adenoid cystic carcinoma (ACC) is a rare, malignant tumor, which usually originates from the minor salivary glands. The laryngeal variant, arising from the glandular components of the larynx, is extremely atypical [1]. A review of the literature on ACC demonstrates only 15 cases reported in the past 40 years in a compressive analysis of the topic [2]. Another review interrogated 1342 cases of laryngeal tumors and identified five cases of ACC [3]. The management for both the laryngeal and glandular components consists of surgical resection and ionizing radiation, which is aimed to achieve local control. However, these modalities do not seem to affect mortality, which is typically the result of metastatic disease [2,4]. Prophylactic lymph node dissection is only indicated for clinically involved nodes [5,6]. ACC is characterized by an indolent growth pattern and late distant metastasis, most commonly to the lungs [2]. Owing to the slow growth and indolent nature of this malignancy, survival for patients with laryngeal ACC is measured in decades [2,5]. Thus, in contrast to other malignancies, ACC survival is typically not measured as five-year mortality, but more commonly at 10 or 20 years [7]. Pulmonary metastasectomies have been reported for isolated lesions from ACC [8]. Glandular ACC has a predilection for the lungs, but has also been reported to metastasize to other organs including brain, bone, liver, thyroid and spleen [9]. The natural history of salivary gland ACC indicates that average time between diagnosis of the primary tumor and death was 60.1 months and the interval between occurrence of metastases and death was 33.0 months [9].

In the present discussion, we address the need to perform a splenectomy in an unusual clinical situation of a metastatic lesion from ACC. We present a review of the literature on metastatic lesions to the spleen and the role of splenectomy for their management.

## Case presentation

A 26-year-old Caucasian man was referred to the surgical service with an enlarging splenic mass and left upper quadrant pain. The pain was described as dull with no exacerbating factors. He had no nausea or vomiting and no other systemic complaints. He had a history of laryngeal ACC that was treated with total laryngectomy and adjuvant radiation three years prior to this clinical visit. Within a year of the original diagnosis and treatment of ACC, our patient developed a single right lung metastasis for which he underwent thoracotomy with resection. This was followed by recurrence in the ipsilateral lung, which was again resected. He was started on Tarceva (erlotinib), an EGFR inhibitor used to treat non-small cell lung cancer, but the therapy was discontinued secondary to an intolerable rash.

**Figure 1:**
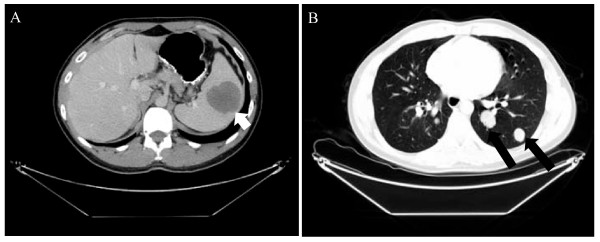
Computed tomography revealed splenic lesion (A, identified with arrow) and demonstrating metastatic lung lesions (B, identified with arrows).

**Figure 2:**
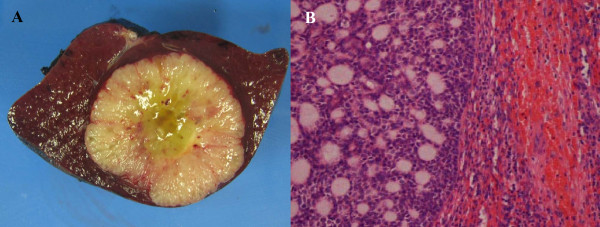
Gross pathologic specimen (A), and microscopic view demonstrating characteristic cribriform pattern abutting splenic capsule (B).

During follow up a computed tomography (CT) scan of the chest demonstrated bilateral lung nodules, which were consistent with metastatic disease (Figure 1B). However, there were no symptoms attributable to these lesions. The CT scan also demonstrated a lesion in the spleen that measured 4cm and was approximately 1mm from the splenic capsule (Figure 1A). He was referred to the surgical service when a follow up CT scan of the abdomen three months later demonstrated progression in size of the lesion by 1cm and our patient complained of left upper quadrant abdominal discomfort. There was no evidence of other intra-abdominal disease. Due to the progression in size of the splenic lesion, its proximity to the capsule with potential complications of rupture or local advancement, and in light of our patient's symptoms the decision was made to proceed with laparoscopic splenectomy. He was given appropriate pre-operative immunization. At the time of surgery, there was no evidence of other intra-abdominal disease. The spleen (Figure 2A) was removed intact through an enlarged port site incision. Pathologic examination (Figure 2B) showed a 5.2 × 4.3 × 4cm single metastatic nodule within 0.1cm from the capsule. The mass shows mucoid areas in the center and microscopically had features characteristic of adenoid cystic carcinoma with a predominant cribriform growth pattern. He had a routine convalescence, and is currently alive and well.

## Discussion

**Figure 3:**
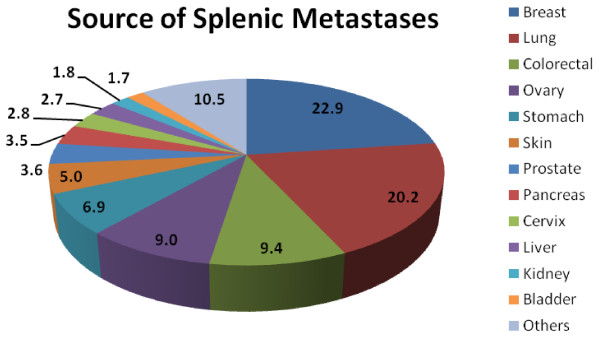
**Chart showing the source of splenic metastases from a large autopsy review**[12].

**Figure 4:**
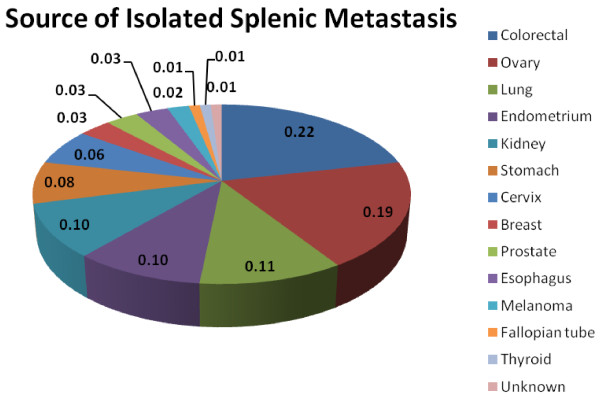
**Chart demonstrating source of solitary splenic metastasis**[13].

Involvement of the spleen as a result of metastatic disease is an unusual event, which occurs in 0.96% to 7.1% of patients with carcinoma as demonstrated by autopsy examinations [10,11]. Splenic metastases often herald late stage disease and are typically associated with multi-visceral metastatic disease. The organ of origin is most commonly breast, followed by lung, colorectal, ovary, stomach and skin (Figure 3) [12]. Isolated splenic metastasis is an even more atypical condition. A recent review of the literature reported only 93 cases of solitary splenic metastasis [13]. The majority of metastases were of colorectal and ovarian origin (Figure 4) [13]. The increased incidence of ovarian splenic metastases may be related to the route of spread (peritoneal seeding versus blood borne metastases) and the close radiographic and clinical follow-up that these patients undergo.

Splenectomy for metastases has been previously reported (Table 1). The most common indication for splenectomy is as part of cytoreductive therapy in patients with ovarian cancer as this might increase the therapeutic efficacy of systemic chemotherapy [14-16]. A splenectomy for the management of colon cancer and melanoma has also been successfully performed as a disease control strategy in the management of these malignancies [17,18]. The cases presented in Table 1 indicate the potential therapeutic benefit of splenectomy as the average post-operative survival for these patients is from 11.1 to 29.3 months.

**Table 1:** Splenectomy for metastatic disease.

*Reference*	***de wilt *** [17]	***Lee *** [15]	***Nicklin *** [16]	***Gemignani *** [14]	***Sileri *** [18]	***Agha-Mohammadi *** [19]
Number	15	31	18	6	16	54
Age	52.7 (37-84)	61 (39-79)	58.1 (30-78)	59 (58-60)	65 (33-81)	n/a
M:f	6.5:1	01:09.3	0:18	0:06	1.8:1	n/a
Primary tumor						
Gynecologic	0	23	14	6	0	33
Colorectal	0	2	0	0	16	8
Melanoma	15	0	0	0	0	1
Lung	0	1	0	0	0	5
Peritoneum		1	3	0	0	0
Other	0	4	1	0	0	7
Solitary lesion	6/15	n/a	n/a	n/a	16/16	n/a
Survival in months (range)	11.3 (1-31)	22.9 (1-72)	11 dead @ 12 (5-59.5)	NED @ 28 (6-65)	not reported	31 NED @ 29.1 (6-144)
			5 alive with disease			5 with disease @ 20.2 (6-72)
			2 NED			6 dead
						12 unknown

The present report is unique in its presentation of laryngeal ACC metastatic to the spleen. This case also illustrates the need to proceed with splenectomy for the management of symptoms and also to prevent substantial adverse outcomes that might result from further tumor involvement. Because of the indolent nature of ACC, a splenic lesion might achieve substantial growth, which if left untreated might rupture causing lethal hemorrhage or erode into the adjacent structures (*i.e*. diaphragm) causing significant symptoms and morbidity.

## Conclusions

Splenic metastasis is a rare event. When it occurs, splenic metastasis is usually associated with widespread metastatic disease. Splenectomy may be considered for patients with isolated disease, patients needing cytoreduction prior to adjuvant therapy, and for those patients with symptomatic disease.

## Consent

Written informed consent was obtained from the patient for publication of this case report and any accompanying images. A copy of the written consent is available for review by the Editor-in-Chief of this journal.

## Competing interests

The authors declare that they have no competing interests.

## Authors' contributions

BM was involved in patient evaluation and management. He is the primary author. TL was involved in patient data collection. AM was the supervising surgeon for the case and was involved with editing the article. SH was involved in editing the article. All authors read and approved the final manuscript.
